# In Situ Grown 1D/2D Structure of Dy_3_Si_2_C_2_ on SiC_w_ for Enhanced Electromagnetic Wave Absorption

**DOI:** 10.3390/ma16093455

**Published:** 2023-04-28

**Authors:** Gang Qin, Yang Li, Wei Zhou, Huidong Xu, Fang Hu, Xiaobing Zhou

**Affiliations:** 1 School of Materials Science and Chemical Engineering, Ningbo University, Ningbo 315211, China; qingang@nimte.ac.cn; 2 Engineering Laboratory of Advanced Energy Materials, Ningbo Institute of Materials Technology and Engineering, Chinese Academy of Sciences, Ningbo 315201, China; xuhuidong@nimte.ac.cn; 3 National Key Laboratory of Science and Technology on High-Strength Structural Materials, Central South University, Changsha 410083, China; liyang16@csu.edu.cn; 4 Hunan Key Laboratory of Applied Environmental Photocatalysis, Changsha University, Changsha 410022, China; zhouwei_csu@163.com

**Keywords:** SiC whisker, Dy_3_Si_2_C_2_, electromagnetic wave absorption, molten salt method

## Abstract

To improve electromagnetic wave (EMW) absorption performance, a novel nano-laminated Dy_3_Si_2_C_2_ coating was successfully in situ coated on the surface of SiC whisker (SiC_w_/Dy_3_Si_2_C_2_) using a molten salt approach. A labyrinthine three-dimensional (3D) net was constructed by the one-dimensional (1D) SiC_w_ coated with the two-dimensional (2D) Dy_3_Si_2_C_2_ layer with a thickness of ~100 nm, which significantly improved the EMW absorption properties of SiC_w_. Compared to pure SiC_w_ with the minimum reflection loss (RL_min_) value of −10.64 dB and the effective absorption bandwidth (EAB) of 1.04 GHz for the sample with a thickness of 4.5 mm, SiC_w_/Dy_3_Si_2_C_2_ showed a significantly better EMW absorption performance with RL_min_ of −32.09 dB and wider EAB of 3.76 GHz for thinner samples with a thickness of 1.76 mm. The enhancement of the EMW absorption performance could be ascribed to the improvement of impedance matching, enhanced conductance loss, interfacial polarization as well as multiple scattering. The SiC_w_/Dy_3_Si_2_C_2_ can be a candidate for EMW absorber applications due to its excellent EMW absorption performance and wide EAB for relatively thin samples, light weight, as well as potential oxidation and corrosion resistance at high temperatures.

## 1. Introduction

Electromagnetic wave (EMW) radiation pollution seriously endangers human health, as a consequence of the widespread applications of the high frequency electronic devices [1,2,3,4,5]. In recent years, numerous EMW absorption materials have been developed to solve these problems [6,7], including carbon-based materials [8,9], magnetic metal materials [10,11,12], ferrite and its composites [13,14,15], and polymer matrix composites [16,17,18]. However, the poor oxidation resistance of carbon-based materials and polymer matrix composites at high temperatures has impeded their applications, despite their excellent EMW absorption properties [19]. Magnetic materials also cannot be used at high temperatures due to the demagnetization [20]. Furthermore, a relatively high density of ferrite materials also hinders their applications in some special fields, such as aerospace. Therefore, the development of high performance EMW absorption materials with high absorption capability, broad effective absorption bandwidth (EAB), low density as well as small thickness, and excellent oxidation resistance at high temperatures is a critical challenge in this field to minimize EMW radiation pollution.

SiC has been considered a promising candidate for EMW absorbers because it has excellent dielectric properties, high temperature stability, as well as outstanding oxidation and corrosion resistance [21,22]. Most of the works on SiC-based EMW absorption materials have been focused on the SiC nanoparticles (SiCNP), SiC fibers (SiC_f_), SiC nanowires (SiCNWs), and SiC whiskers (SiC_w_) [23,24,25,26,27,28]. Among all of them, one-dimensional (1D) SiC_w_ or SiCNWs have drawn the most significant attention, since they have large aspect ratio, which is good for dissipating current by providing long transport paths, resulting in a strong conduction loss [29]. Furthermore, a three-dimensional (3D) network can be easily constructed, which is beneficial to improving the EMW absorption performance [27,30]. However, the EMW absorption properties of pure SiC_w_ cannot meet the strict requirements of a strong absorption and a broad EAB because of the poor impedance matching and single EMW loss mechanism [31,32,33]. Therefore, many research works have been conducted to improve the EMW absorption properties of SiC_w_, including elemental doping, surface modification, and fabrication of SiC_w_-based composites [34,35]. For example, Kuang et al. reported that the electrical conductivity of SiC_w_ was significantly enhanced by Al-doping. The lowest reflection loss (RL) value was −25.4 dB, and the EAB was 2 GHz when the Al/Si ratio was 0.03/0.97 [29]. In particular, surface modification of SiC_w_ has been demonstrated to be an efficient and feasible method for improving the EMW absorption [27,31,36].

Rare earth silicide carbides (RE_3_Si_2_C_2_, where RE is a rare earth element) are a new group of ternary layered structure materials, which are similar to MAX phases (where M is an early transition metal, A is an A-group element, and X is either C or N) [37,38]. RE_3_Si_2_C_2_ has been successfully used as the joining layer material and/or sintering additive for SiC-based ceramics and composites due to its ability to form a liquid phase by the eutectic reaction with SiC [39,40,41,42,43,44,45,46]. Furthermore, the addition of a second phase can significantly promote the EMW absorption properties of the resulting composites [47,48]. Our previous work indicated that the EMW absorption properties of SiC_f_ can be significantly improved by the incorporation of Y_3_Si_2_C_2_ coating on the SiC_f_ surface [3]. The minimum RL of SiC_f_/Y_3_Si_2_C_2_ was -16.98 dB at a thin thickness of 2.19 mm. Furthermore, compared to the EAB of 1.92 GHz at the thickness of 3.38 mm for pure SiC fiber, SiC_f_/Y_3_Si_2_C_2_ shows significantly wider optimal EAB of 5.44 GHz at a much thinner thickness of 2.64 mm [3]. SiC_w_ whiskers have a larger aspect ratio compared to the chopped SiC fibers. It is relatively easy to form a 3D net, which is beneficial for increasing the heterogeneous interfaces and multiple reflections and scattering. Therefore, it is expected that SiC_w_/Dy_3_Si_2_C_2_ could show even better EMW absorption properties when compared to SiC_f_/Y_3_Si_2_C_2_.

In this work, the two-dimensional (2D) Dy_3_Si_2_C_2_ coating is formed on the one-dimensional (1D) SiC_w_ surface by the molten salt method to improve the EMW absorption properties. Combining Dy_3_Si_2_C_2_ with SiC_w_ not only effectively improved the impedance matching but also provided a large number of heterogeneous interfaces as well as enhanced interface polarization loss. At the same time, the stacking effect of one-dimensional structures builds an efficient three-dimensional conductive network that enhances resistance loss. Furthermore, the two-dimensional layered structure of Dy_3_Si_2_C_2_ can improve multiple reflections, which is beneficial to improving the EMW absorption properties. Microstructure, phase composition, dielectric, and EMW absorption properties of the as-obtained SiC_w_/Dy_3_Si_2_C_2_ coated whiskers were investigated. The possible EMW absorption mechanism of SiC_w_/Dy_3_Si_2_C_2_ was summarized. EMW absorption properties of the as-obtained SiC_w_/Dy_3_Si_2_C_2_ were compared to the previously reported materials.

## 2. Experimental Procedure

### 2.1. Materials and Experiments

DyH_2_ powder with a purity of 99.9% and a mean particle size of ~75 μm was purchased from Institute of Hunan rare earth metal materials Co., Ltd., Changsha, China. SiC whiskers (Union Materials Co., Daegu, Republic of Korea) with a diameter of 0.4–0.9 μm and a length of 6–120 μm; NaCl; and KCl powders (purity: 99.5%, mean particle size: 75 µm; Sinopharm Chemical Reagent Co., Ltd., Shanghai, China) were used as the raw materials.

The DyH_2_, SiC_w_, NaCl, and KCl powders with the molar ratio of DyH_2_: SiC_w_ =1:4 and NaCl:KCl = 1:1 were mixed for 30 min in an Ar atmosphere in a home-made glove box. The mixed DyH_2_, SiC_w_, NaCl, and KCl powders were heated to a target temperature of 1000 °C in the molten salt furnace. The holding time was set 5 h. The heating and cooling rate was 5 °C/min. The as-obtained samples were washed and filtered using deionized water several times. The in situ coated SiC_w_/Dy_3_Si_2_C_2_ powder can be obtained after drying 12 h at 60 °C in a vacuum oven.

### 2.2. Characterizations

The phase compositions of the as-obtained SiC_w_/Dy_3_Si_2_C_2_ were detected using an X-ray diffractometer (XRD: D8 Advance, Bruker AXS, Karlsruhe, Germany) using Cu Kα radiation (λ = 1.5406 Å). The operating current and voltage were 40 mA and 40 kV, respectively. The step scan and step time was 0.02° 2θ and 0.2 s, respectively. The microstructure of the SiC_w_/Dy_3_Si_2_C_2_ powders was observed using a scanning electron microscope (SEM, 8230, Hitachi, Tokyo, Japan). The microstructure and phase compositions of the Dy_3_Si_2_C_2_ coating were further investigated using a transmission electron microscope (TEM, Talos F200X, Thermo Fisher Scientific, Waltham, MA, USA) equipped with an energy dispersive spectroscopy (EDS) system. The samples for TEM observations were prepared using the focused ion beam (FIB, Auriga, Carl Zeiss, Jena, Germany) technique. The complex permittivity and complex permeability were measured at a frequency range from 2 to 18 GHz using a Network Analyzer of Agilent N5230A. In order to measure the complex permittivity and complex permeability, SiC_w_/Dy_3_Si_2_C_2_ powder was mixed with 50 wt.% paraffin with a size of an inner and outer diameter of 3 and 7 mm as well as a thickness of 2 mm, respectively. For the sake of comparison, the electromagnetic properties of the pure SiC whiskers were detected using the same method.

## 3. Results and Discussion

### 3.1. Microstructure and Phase Composition of SiC_w_/Dy_3_Si_2_C_2_

**Figure 1:**
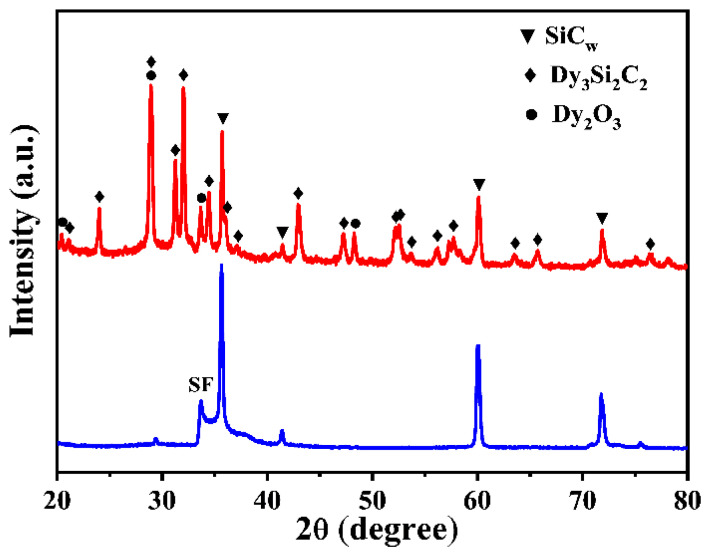
XRD patterns of pure SiC and SiC_w_/Dy_3_Si_2_C_2_ whiskers.

Figure 1 presents the XRD patterns of the pure SiC whiskers and the as-obtained SiC_w_/Dy_3_Si_2_C_2_ whiskers.

The XRD pattern of pure SiC whiskers indicated that they are formed by the 3C-SiC phase (JCPDS No. 75-0254). A small peak at approximately 33.5° corresponds to the stacking faults, which spontaneously formed during the growing process of SiC whiskers. The XRD pattern of the SiC_w_/Dy_3_Si_2_C_2_ powder revealed that besides SiC_w_, it also contained characteristic peaks of Dy_3_Si_2_C_2_ (JCPDS No. 97-005-1299) along with some impurities of Dy_2_O_3_ (JCPDS No. 97-018-5606). This confirmed that the Dy_3_Si_2_C_2_ modification of SiC whiskers was successfully obtained.

**Figure 2:**
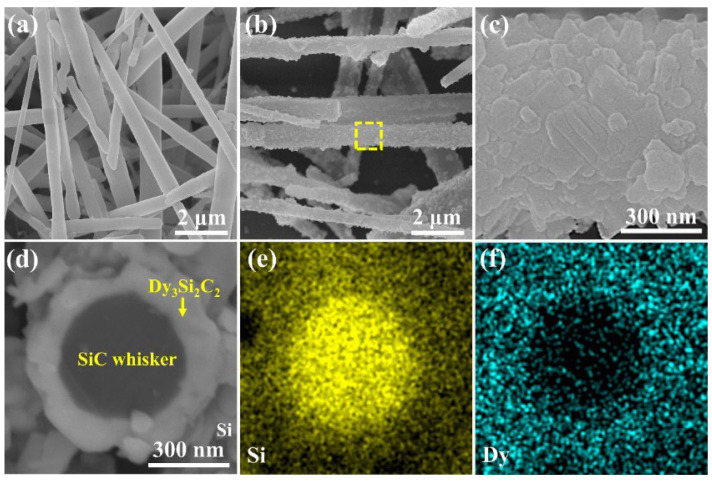
SEM images of pure SiC_w_ (**a**) and SiC_w_/Dy_3_Si_2_C_2_ (**b**,**c**), (**c**) is the high magnification SEM image of the yellow dashed area in (**b**), cross-section back scattered SEM image of SiC_w_/Dy_3_Si_2_C_2_ (**d**) and its corresponding EDS elemental distribution of Si (**e**) and Dy (**f**).

Figure 2 shows the SEM images of both SiC_w_ and SiC_w_/Dy_3_Si_2_C_2_ whiskers.

The diameter of the pure SiC whiskers was ~500 nm. A dense 2D structure Dy_3_Si_2_C_2_ coating with a structure of randomly oriented nano-laminated sheets was in situ coated on the surface of SiC whiskers (Figure 2b,c). The thickness of Dy_3_Si_2_C_2_ coating was around 100 nm, as shown in the SEM image of the fracture surface of the SiC_w_/Dy_3_Si_2_C_2_ whisker (Figure 2d). The corresponding elemental distribution of Si and Dy indicated that most of the Dy_3_Si_2_C_2_ was homogenously coated on the surface of SiC whisker (Figure 2e,f).

**Figure 3:**
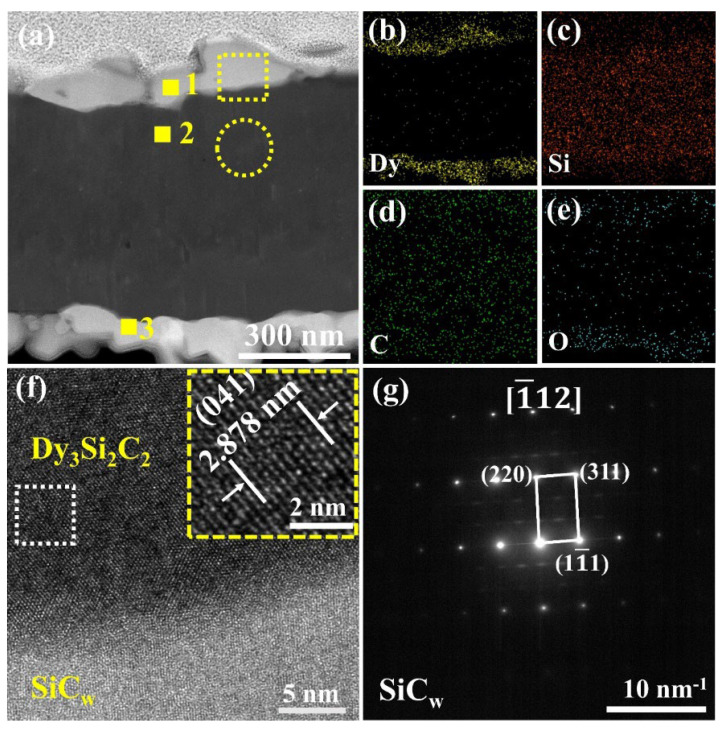
(**a**) HAADF image of the as-synthesized SiC_w_/Dy_3_Si_2_C_2_ and the corresponding EDS mapping of (**b**) Dy, (**c**) Si, (**d**) C, and (**e**) O, (**f**) HR−TEM image of the Dy_3_Si_2_C_2_ coating, as highlighted by a yellow rectangle in (**a**), and (**g**) the SAED pattern taken from the area highlighted by the yellow circle in (**a**).

To further confirm the microstructure and phase composition of the Dy_3_Si_2_C_2_ coating, semi-quantitative EDS and high-resolution transmission electron microscope (HR-TEM) analysis along with selected-area electron diffraction (SAED) were performed. Figure 3 presents a high-angle annular dark-field (HAADF) image of the as-synthesized SiC_w_/Dy_3_Si_2_C_2_ and the corresponding Dy, Si, C, and O elemental distributions, respectively.

The semiquantitative EDS analysis results of areas 1–3 are shown in Table 1, suggesting the presence of SiC, Dy_3_Si_2_C_2_, and/or Dy_2_O_3_.

Furthermore, the HR-TEM image of the interface between SiC_w_ and Dy_3_Si_2_C_2_ coating is shown in Figure 3f. The lattice fringe spacing was 0.2878 nm, which can be assigned to the (041) planes of Dy_3_Si_2_C_2_. Therefore, taking into account all the results obtained by XRD, EDS, and HR-TEM analysis, it can be concluded that a dense ~100 nm Dy_3_Si_2_C_2_ coating was successfully fabricated on the surface of SiC_w_ using the molten salt approach.

The formation process of the Dy_3_Si_2_C_2_ coating using the molten salt approach is similar to the formation mechanism of Y_3_Si_2_C_2_ and Pr_3_Si_2_C_2_ powders [45,48]. First, DyH_2_ decomposed to Dy and released H_2_ [49]. The Dy element diffused to the surface of SiC whiskers via the liquid molten salt, and then the Dy_3_Si_2_C_2_ coating was formed. The main reactions can be summarized as follow:

(1) $${\mathrm{DyH}}_{2}\to \mathrm{Dy}+H_{2}$$

(2) $$3\mathrm{Dy}+2\mathrm{SiC}\to {\mathrm{Dy}}_{3}{\mathrm{Si}}_{2}C_{2}$$

On the other hand, the potential formation barrier of the Dy_3_Si_2_C_2_ could decline because the surface energy of both DyH_2_ and SiC_w_ could be remarkably promoted by polarization effect of the molten salt [50,51]. In addition, the diffusion rate of the Dy, Si, and C atoms can be obviously promoted in the liquid molten salt reaction medium. Therefore, the Dy_3_Si_2_C_2_ coating can be in situ formed on the surface of SiC whiskers at a relatively low temperature (1000 °C) and adhered well to the surface of SiC_w_.

### 3.2. Dielectric Properties of SiC_w_/Dy_3_Si_2_C_2_

**Figure 4:**
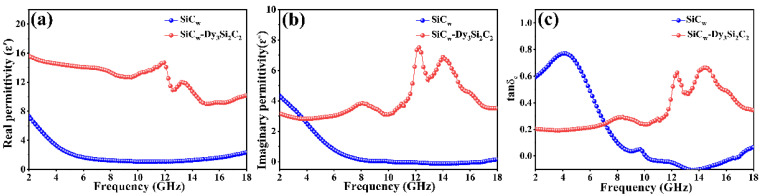
Real (**a**) and imaginary (**b**) parts of the complex permittivity, as well as the loss angle (**c**) of the pure SiC_w_ and SiC_w_/Dy_3_Si_2_C_2_ whiskers.

The EMW absorption property of materials is mainly confirmed by their complex permittivity and permeability. Meanwhile, good impedance matching between absorbing materials and free space can make EMW incident into materials with less reflection. While SiC_w_ and SiC_w_/Dy_3_Si_2_C_2_ are nonmagnetic materials, the real (μ′) and imaginary (μ″) parts of the complex permeability is around 1 and 0, respectively (not shown here). Therefore, the EMW absorption capability of SiC_w_ and SiC_w_/Dy_3_Si_2_C_2_ is highly dependent on their complex permittivity. The real (*ε*′) and imaginary (*ε*″) parts of complex permittivity of the pure SiC_w_ and SiC_w_/Dy_3_Si_2_C_2_ whiskers are shown in Figure 4.

Most of the real (*ε*′) and imaginary (*ε*″) parts of the complex permittivity of SiC_w_/Dy_3_Si_2_C_2_ were higher than that of pure SiC_w_, indicating that the Dy_3_Si_2_C_2_ coating could promote the dielectric properties of SiC_w_.

According to the Debye theory, *ε*′ and *ε*″ can be calculated by the following equations [52]:

(3) $$\varepsilon^{\prime }=\varepsilon_{\infty }+\left(\varepsilon_{s}-\varepsilon_{\infty }\right)/\left(1+{\left(\omega \tau \right)}^{2}\right)$$

(4) $$\varepsilon^{\prime \prime }=\left(\varepsilon_{s}-\varepsilon_{\infty }\right)/\left(1+{\left(\omega \tau \right)}^{2}\right)+\delta_{ac}/\omega \varepsilon_{0}={\varepsilon_{p}}^{\prime \prime }+{\varepsilon_{c}}^{\prime \prime }$$

where *ε*_0_, *ε_s_*, and $\varepsilon_{\infty }$ represent free space dielectric constant, the permittivity in static state, and light frequency, respectively. *ω* and *τ* are angular frequency and polarization relaxation time, respectively. *σ* is electric conductivity. *ε_p_*″ and *ε_c_*″ correspond to the contributions to *ε*″ from polarization loss and conductance loss, which are associated with *σ*. Generally, the real part of the permittivity signifies the storage capability of the dielectric energy, while the imaginary part of the permittivity stands for the loss of dielectric energy [53]. Thus, the improvement of *ε*′ can be ascribed to the interfacial polarization caused by the improved heterogeneous interfaces in SiC_w_/Dy_3_Si_2_C_2_ whiskers, which were generated by the incorporation of nano-laminated (2D) Dy_3_Si_2_C_2_ coating on the surface of SiC_w_. The enhancement of *ε*″ was mainly decided by the increasing of the electrical conductivity ( σ ), where σ can be confirmed by the follow equation [54]:

(5) $$\sigma =2\pi \varepsilon_{0}\varepsilon \varepsilon^{\prime \prime }$$

where *ε*_0_ represents the permittivity in a vacuum.

**Figure 5:**
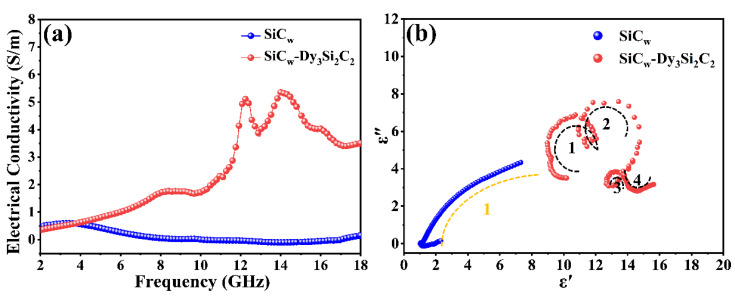
Electrical conductivity (**a**,**b**) Cole–Cole curves of the pure SiC_w_ and SiC_w_/Dy_3_Si_2_C_2_.

The electrical conductivity of SiC_w_/Dy_3_Si_2_C_2_ was higher than that of pure SiC_w_, as shown in Figure 5a.

This can be mainly attributed to the metallic conductivity characteristic of the 2D structural Dy_3_Si_2_C_2_ coating, as the coating formed a net structure, increasing the transmission channels of carriers [55]. In addition, both *ε*′ and *ε*″ of SiC_w_/Dy_3_Si_2_C_2_ showed a fluctuation corresponding to the resonance, while this was not observed for the pure SiC_w_. The permittivity of the SiC_w_/Dy_3_Si_2_C_2_ whiskers showed typical nonlinear resonant characteristics, indicating the existence of polarization and relaxation behavior, which implied better dielectric loss performance in the corresponding frequency range. The Cole–Cole semicircle was used to investigate the relaxation polarization process. According to the Debye theory, the relationship between *ε*′ and *ε*″ can be expressed by Equation (6) [56]:

(6) $${\left(\varepsilon^{\prime \prime }-\left(\varepsilon_{s}+\varepsilon_{\infty }\right)/2\right)}^{2}+\varepsilon^{\prime \prime }=\left(\varepsilon_{s}-\varepsilon_{\infty }\right)/2$$

The Cole–Cole curves of pure SiC_w_ and SiC_w_/Dy_3_Si_2_C_2_ are shown in Figure 5b. Each Deby relaxation process is manifested by one Cole-Cloe semicircle [55,56]. There was only one Cole–Cole semicircle observed in pure SiC_w_, indicating one relaxation process, while four semicircles were observed in SiC_w_/Dy_3_Si_2_C_2_, confirming the improvement of dielectric loss capacity in SiC_w_/Dy_3_Si_2_C_2_. The improvement of the relaxation process of SiC_w_/Dy_3_Si_2_C_2_ was mainly caused by the significantly increased interface relaxation, which resulted from the improved number of heterogeneous interfaces in SiC_w_/Dy_3_Si_2_C_2_.

### 3.3. Electromagnetic Wave Absorption Performance

Reflection loss (RL) and effective absorption bandwidth (EAB, the corresponding frequency range of RL < −10 dB, which presents more than 90% EMW energy absorbed) are usually used to evaluate the EMW absorption performance of materials. According to the transmission line theory, the RL values of SiC_w_ and SiC_w_/Dy_3_Si_2_C_2_ can be calculated by the following equations [57,58,59]:

(7) $$RL\left(dB\right)=20\log\left|\left(Z_{in}-Z_{0}\right)/\left(Z_{in}+Z_{0}\right)\right|$$

(8) $$Z_{in}=Z_{0}\sqrt{\mu_{r}/\varepsilon_{r}}\tanh\left[j\left(2\pi fd/c\right)\sqrt{\mu_{r}\varepsilon_{r}}\right]$$

(9) $$Z_{0}=\sqrt{\mu_{r}/\varepsilon_{r}}$$

where *Z*_0_ and *Z_in_* is space free impedance and input impedance, respectively. *c*, *d*, and f are speed of light, thickness, and frequency, respectively. *μ_r_* = *μ′* − *jμ*″ and *ε_r_* = *ε*′ − *jε*″ represent the complex permeability and permittivity of material.

**Figure 6:**
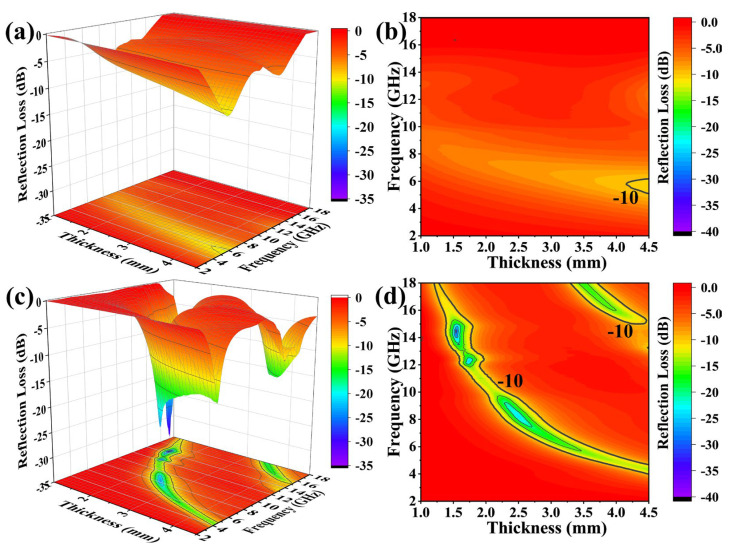
3D and 2D patterns of RL values at the frequency range of 2 to 18 GHz for different thicknesses of SiC_w_ (**a**,**b**) and SiC_w_−Dy_3_Si_2_C_2_ samples (**c**,**d**).

Figure 6 shows the 3D and 2D plots of RL values at the frequency range of 2 to 18 GHz at different thicknesses of the SiC_w_ and SiC_w_/Dy_3_Si_2_C_2_ samples.

**Figure 7:**
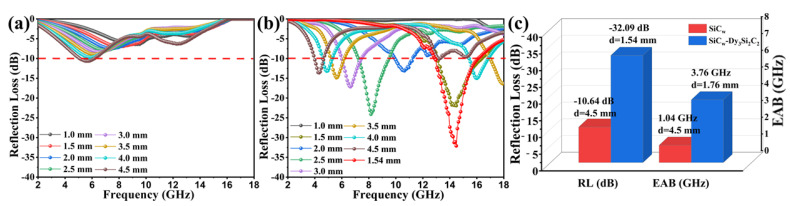
RE values at the frequency range of 2 to 18 GHz for different thicknesses of the SiC_w_ (**a**) and SiC_w_/Dy_3_Si_2_C_2_ samples (**b**), (**c**) comparison of the RL_min_ and EAB of the pure SiC_w_ and SiC_w_−Dy_3_Si_2_C_2_ samples.

The minimum RL (RL_min_) value of the pure SiC_w_ is −10.64 dB at the frequency of 5.52 GHz with the 4.5 mm sample thickness. After coating of SiC_w_ with 2D Dy_3_Si_2_C_2_ sheets, the RL_min_ value was improved to −32.09 dB at the frequency of 14.48 GHz for the 1.54 mm sample thickness. For convenience of comparison, the selected theoretical calculated RL of pure SiC_w_ and SiC_w_/Dy_3_Si_2_C_2_ with different thicknesses in the frequency range of 2 to 18 GHz is shown in Figure 7a,b.

It is obvious that the EAB of SiC_w_/Dy_3_Si_2_C_2_ is much wider than that of SiC_w_ for the samples with the thickness range of 1 to 4.5 mm at the frequency ranging from 2 to 18 GHz. The widest EAB can be as high as 3.76 GHz for thin SiC_w_/Dy_3_Si_2_C_2_ samples with the thickness of 1.76 mm (Figure 7c). However, the widest EAB of pure SiC_w_ is only 1.04 GHz for the sample with a thickness of 4.5 mm (Figure 7c). This indicates that the Dy_3_Si_2_C_2_ coating can significantly improve the EMW absorption properties of SiC_w_.

In order to reveal the intrinsic reason for the improved EMW absorption performance for SiC_w_/Dy_3_Si_2_C_2_, the impedance match (*Z*) as well as the attenuation constant (α) were calculated. *Z* was confirmed by the following equation [60]:

(10) $$Z=\left|Z_{in}/Z_{0}\right|=\sqrt{\mu_{r}/\varepsilon_{r}}\tanh\left[j\left(2\pi fd/c\right)\sqrt{\mu_{r}\varepsilon_{r}}\right]$$

**Figure 8:**
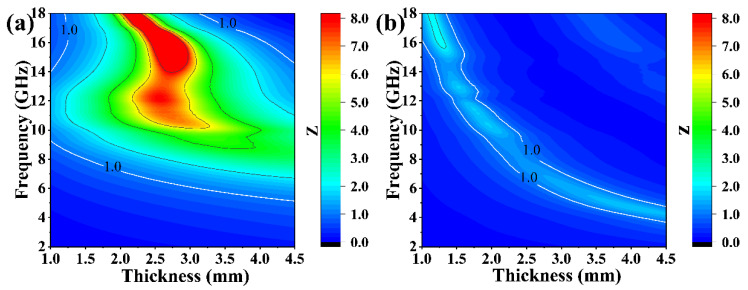
2D patterns of Z value of SiC_w_ (**a**) and SiC_w_/Dy_3_Si_2_C_2_ (**b**).

A favorable impedance match is the basic requirement to obtain an excellent EMW absorption performance, which ensures the EMW can enter materials instead of being reflected [61,62,63,64]. According to Equation (10), when the input impedance (*Z_in_*) is infinitely close to the air impedance (*Z*_0_), the ideal impedance matching can be obtained. Figure 8a,b presents the calculated *Z* values of the pure SiC_w_ and SiC_w_/Dy_3_Si_2_C_2_ samples with the thickness of 1–4.5 mm at the frequency ranging from 2 to 18 GHz.

The frequency range with good impedance match (Z-value is close to 1) of SiC_w_/Dy_3_Si_2_C_2_ was much larger than that of pure SiC_w_, which indicates that the impedance match of the SiC_w_ was well improved by the Dy_3_Si_2_C_2_ coating. Therefore, the EMW can enter the SiC_w_/Dy_3_Si_2_C_2_ sample, while most of the EMW was reflected in the case of pure SiC_w_ due to the poor impedance matching.

**Figure 9:**
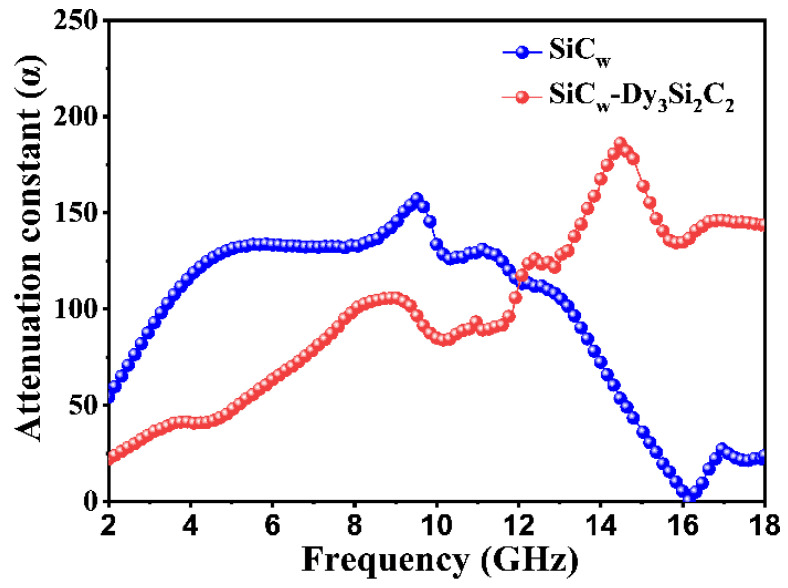
Attenuation constant of pure SiC_w_ and SiC_w_/Dy_3_Si_2_C_2_ at the frequency range from 2 to 18 GHz.

Furthermore, to evaluate the attenuation ability of EMW energy of the samples, the α (Figure 9) was evaluated by the following formula [53]:

(11) $$\alpha =\frac{\sqrt{2}\pi f}{c}\sqrt{\left(\mu^{\prime \prime }\varepsilon^{\prime \prime }-\mu^{\prime }\varepsilon^{\prime }\right)+\sqrt{{\left(\mu^{\prime \prime }\varepsilon^{\prime \prime }-\mu^{\prime }\mu^{\prime }\right)}^{2}+{\left(\mu^{\prime }\varepsilon^{\prime \prime }-\mu^{\prime \prime }\varepsilon^{\prime }\right)}^{2}}}$$

A larger value of α implies a stronger attenuation ability [65]. The sole high dielectric loss of SiC_w_ at a low frequency resulted in a high attenuation constant, which meant that most of the EMW was reflected. This is in good agreement with the poor impedance matching of the SiC_w_ sample. On the other hand, the introduction of the nano-laminated (2D) Dy_3_Si_2_C_2_ coating significantly improved the impedance match as well as the attenuation ability of the SiC_w_/Dy_3_Si_2_C_2_. As a result, the EMW absorption property was significantly improved.

**Figure 10:**
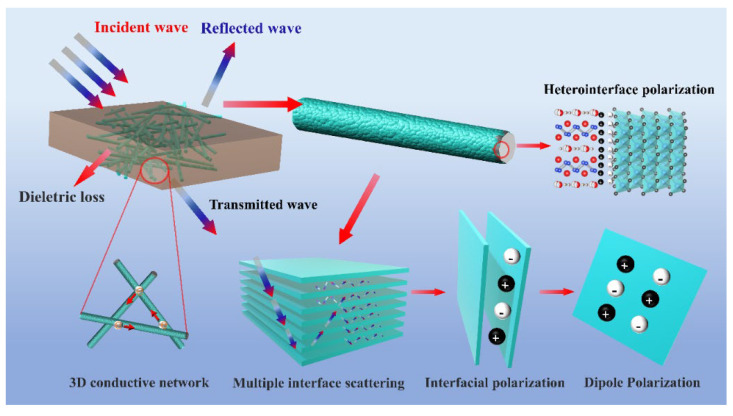
The EMW absorption mechanism of SiC_w_/Dy_3_Si_2_C_2_.

The possible EMW absorption mechanism of SiC_w_/Dy_3_Si_2_C_2_ is illustrated in Figure 10.

Firstly, the favorable impedance matching suggests that the majority of the EMW can enter the SiC_w_/Dy_3_Si_2_C_2_ sample, while just a small part of the EMW is reflected. This is the premise of excellent EMW absorption performance of the material. Secondly, the metallic conductivity characteristic of Dy_3_Si_2_C_2_ coating improved the electrical conductivity of SiC_w_/Dy_3_Si_2_C_2_, which enhanced the conductance loss by improving the electron transition channel in SiC_w_/Dy_3_Si_2_C_2_. Thirdly, a large number of heterogeneous interfaces in the SiC_w_/Dy_3_Si_2_C_2_ sample, such as Dy_3_Si_2_C_2_/Dy_3_Si_2_C_2_, SiC_w_/Dy_3_Si_2_C_2_, and SiC_w_/SiC_w_, significantly increased the interfacial polarization and hopping electrons between Dy_3_Si_2_C_2_ nanosheets. This is beneficial for the improvement of the dielectric loss of the material. Finally, the high aspect ratio of SiC_w_ with the 2D nano-laminated Dy_3_Si_2_C_2_ coating constructed a 3D microstructure and formed an effective conductive network, resulting in the enhancement of multiple scattering and reflections. Therefore, the excellent EMW absorption performance of SiC_w_/Dy_3_Si_2_C_2_ was attributed to the synergistic effect of favorable impedance matching, enhanced conductance loss, interfacial polarization, dipole polarization, and multiple scattering and reflections.

**Figure 11:**
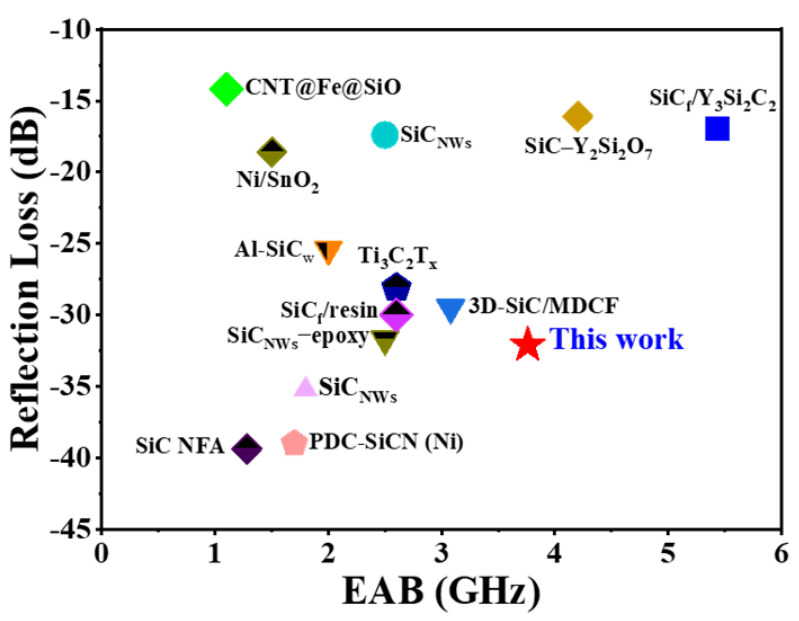
The comparison of EMW absorption properties of SiC_w_/Dy_3_Si_2_C_2_ with other materials [3,23,29,66,67,68,69,70,71,72,73,74,75].

The EMW absorption property of SiC_w_/Dy_3_Si_2_C_2_ is better when compared to most of the previously reported materials, as shown in Figure 11.

It can be concluded that the as-obtained SiC_w_/Dy_3_Si_2_C_2_ whiskers could be a promising candidate for EMW absorbers for aerospace applications due to their excellent EMW absorption performance and wide EAB for thin samples, light weight, and potential oxidation resistance at high temperatures.

## 4. Conclusions

In summary, a novel nano-laminated Dy_3_Si_2_C_2_ coating was in situ fabricated on the surface of SiC_w_ using the molten salt method to improve EMW absorption performance. A randomly stacked 2D Dy_3_Si_2_C_2_ nanosheet coating with a thickness of ~100 nm was uniformly coated on the surface of 1D SiC_w_, which further formed a 3D microstructure. The EMW absorption performance of the as-obtained 3D structural SiC_w_/Dy_3_Si_2_C_2_ sample was significantly improved when compared to the pure SiC_w_ sample. The minimum RL value increased from −10.64 dB for the pure SiC_w_ to −32.09 dB for the SiC_w_/Dy_3_Si_2_C_2_. At the same time, the corresponding thickness of 1.54 mm was much thinner than that of the pure SiC_w_ (4.5 mm). The possible EMW absorption mechanism of the as-obtained SiC_w_/Dy_3_Si_2_C_2_ sample was ascribed to the synergic effect of favorable impedance matching, enhanced conductance loss, interfacial polarization, dipole polarization, and multiple scattering. The as-obtained 3D structural SiC_w_/Dy_3_Si_2_C_2_ could be a candidate for EMW absorber applications due to its excellent EMW absorption performance and wide EAB for relatively thin samples, light weight, as well as potential oxidation and corrosion resistance at high temperatures.

## Figures and Tables

**Table 1:** EDS results collected from points 1–3 in Figure 3a.

No.	Composition in Atomic %				Probable Phases
	Dy	Si	C	O	
1	45.92	23.11	15.72	15.25	Dy_3_Si_2_C_2_, Dy_2_O_3_
2	0.39	59.29	36.12	4.20	SiC_w_
3	42.11	22.98	16.99	17.93	Dy_3_Si_2_C_2_, Dy_2_O_3_

## Data Availability

All research data already shared in the manuscript.
